# Effects of docosahexaenoic acid and eicosapentaoic acid supplementation on white matter integrity after repetitive sub-concussive head impacts during American football: Exploratory neuroimaging findings from a pilot RCT

**DOI:** 10.3389/fneur.2022.891531

**Published:** 2022-09-15

**Authors:** Adam C. Raikes, Gerson D. Hernandez, Veronica A. Mullins, Yiwei Wang, Claudia Lopez, William D. S. Killgore, Floyd H. Chilton, Roberta D. Brinton

**Affiliations:** ^1^Center for Innovation in Brain Science, University of Arizona, Tucson, AZ, United States; ^2^Department of Nutritional Sciences, University of Arizona, Tucson, AZ, United States; ^3^Social, Cognitive, and Affective Neuroscience Lab, Department of Psychiatry, College of Medicine, University of Arizona, Tucson, AZ, United States

**Keywords:** sub-concussive impact, football, docosahexaenoic acid, diffusion tensor imaging, functional connectivity

## Abstract

**Context:**

Repetitive sub-concussive head impacts (RSHIs) are common in American football and result in changes to the microstructural integrity of white matter. Both docosahexaenoic acid (DHA) and eicosapentaoic acid (EPA) supplementation exerted neuroprotective effects against RSHIs in animal models and in a prior study in football players supplemented with DHA alone.

**Objective:**

Here, we present exploratory neuroimaging outcomes from a randomized controlled trial of DHA + EPA supplementation in American football players. We hypothesized that supplementation would result in less white matter integrity loss on diffusion weighted imaging over the season.

**Design, setting, participants:**

We conducted a double-blind placebo-controlled trial in 38 American football players between June 2019 and January 2020.

**Intervention:**

Participants were randomized to the treatment (2.442 g/day DHA and 1.020 g/day EPA) or placebo group for five times-per-week supplementation for 7 months. Of these, 27 participants were included in the neuroimaging data analysis (*n* = 16 placebo; *n* = 11 DHA + EPA).

**Exploratory outcome measures:**

Changes in white matter integrity were quantified using both voxelwise diffusion kurtosis scalars and deterministic tractography at baseline and end of season. Additional neuroimaging outcomes included changes in regional gray matter volume as well as intra-regional, edge-wise, and network level functional connectivity. Serum neurofilament light (NfL) provided a peripheral biomarker of axonal damage.

**Results:**

No voxel-wise between-group differences were identified on diffusion tensor metrics. Deterministic tractography using quantitative anisotropy (QA) revealed increased structural connectivity in ascending corticostriatal fibers and decreased connectivity in long association and commissural fibers in the DHA+EPA group compared to the placebo group. Serum NfL increases were correlated with increased mean (ρ = 0.47), axial (ρ = 0.44), and radial (ρ = 0.51) diffusivity and decreased QA (ρ = −0.52) in the corpus callosum and bilateral corona radiata irrespective of treatment group. DHA + EPA supplementation did preserve default mode/frontoparietal control network connectivity (*g* = 0.96, *p* = 0.024).

**Conclusions:**

These exploratory findings did not provide strong evidence that DHA + EPA prevented or protected against axonal damage as quantified *via* neuroimaging. Neuroprotective effects on functional connectivity were observed despite white matter damage. Further studies with larger samples are needed to fully establish the relationship between omega-3 supplementation, RSHIs, and neuroimaging biomarkers.

**Trial registration:**

ClinicalTrials.gov-NCT04796207

## Introduction

Sports-related concussions (SRCs), a subset of broader mild traumatic brain injuries, are a significant public health concern and research emphasis. Recent estimates suggest that over 10,000 SRCs occur each year in organized collegiate sports alone (1). These injuries are associated with short-term (generally resolving within the first month post-injury) somatic symptom presentation (2, 3), disruptions in neurocognitive and motor function (3, 4), oculomotor impairments (3, 4), affective and behavioral changes (5, 6), and disruptions to sleep (7–9). While the majority of these symptoms and impairments resolve quickly, many others persist long after the injurious event (10–12).

While SRCs are common within sport, repetitive sub-concussive head impacts (RSHI) are even more common (13–16). RSHIs are forces imparted to the brain which do not result in the overt signs and functional disruptions associated with a concussion (17). Recent research shows that repetitive head impacts are associated with changes in brain structure (cortical thickness, volume, and microstructural properties of white matter) as well as function (18, 19). The most frequently observed changes are related to white matter microstructure which can occur in as short a time span as a single season and persist after the season ends (20–24). Common findings include reduced fractional anisotropy in long association tracts as well as increased mean and radial diffusivity in the corpus callosum (20–24), which collectively indicate reduced microstructural integrity resulting in less organized diffusion. Notably, findings of this nature are not homogeneous, with some research indicating no or very limited change in white matter integrity indicators (25–27). Given the frequency of RHSIs and the potential for long-term detrimental effect on brain structure, interventions that mitigate the adverse effects of RSHIs could significantly change the course of neurological damage.

One promising preventive intervention is supplementation with omega-3 fatty acids, including docosahexaenoic acid (DHA) and eicosapentaoic acid (EPA). DHA and EPA are long-chain poly-unsaturated fatty acids producing both anti-inflammatory and inflammation-resolving lipid mediators, with noted neuroprotective effects (28). Findings from animal models of traumatic brain injury indicate that DHA supplementation prior to injury improves neurological functioning and cognition, reduces endoplasmic reticulum stress, reduces neuroinflammation and attenuates the expression of amyloid precursor protein and phosphorylated tau after injury (29–33). Additional evidence indicates that supplementation with DHA alone or coupled with other omega-3 fatty acids including EPA initiated after injury exhibits similar effects on amyloid precursor protein, as well as restores energy homeostasis, reduces protein oxidation, and stabilizes cell membrane homeostasis (34–37). However, none of these studies examined the potential preventative neuroprotective role of DHA, in isolation or coupled with other omega-3 fatty acids, against RSHIs.

Two separate clinical trials have indicated that DHA supplementation may be effective at reducing neurological damage from RSHIs (38, 39). Both studies used blood-based biomarkers, specifically neurofilament light (NfL), and reported that American football players – particularly starters – supplemented with DHA throughout the preseason and during the competitive season exhibited less serum NfL compared to placebo-matched controls at the end of a single season, suggesting less accumulated neurological trauma (38, 39). While no prior work has investigated the effects of DHA supplementation on neuroimaging biomarkers in RSHI vulnerable populations, there is evidence of neuroprotective effects from studies of white matter integrity, primarily in the corpus callosum, in other populations including major depression (40), psychosis (41), and attention deficit/hyperactive disorder (42).

Reported herein are exploratory neuroimaging outcomes of a double-blind, randomized, placebo-controlled trial to investigate the potential neuroprotective effect of DHA + EPA supplementation, to capitalize on the observed neuroprotective effects of both of these omega-3 fatty acids, in the context of RSHIs. Primary findings from this trial are reported in Mullins et al. (43) and the analyses here are for a subset of those participants who completed all trial procedures as well as had complete neuroimaging data. Based on previous preclinical and clinical reports regarding the potential of DHA and EPA to reduce the neurological damage from brain injuries (29–37), we utilized the framework presented in Oliver et al. (38) to investigate the impact of DHA + EPA supplementation, rather than DHA alone, on white matter microstructural integrity, quantified via diffusion kurtosis imaging, in males aged 18–27 over the course of a single National Collegiate Athletic Association (NCAA) football season.

## Methods

### Study design and participants

This randomized, double-blinded, placebo-controlled trial was conducted from June 2019 to January 2020 at the University of Arizona. Full eligibility criteria, intervention details, study procedures, and all primary and secondary outcomes are reported in Mullins et al. (43). For consistency with CONSORT reporting guidelines (see Supplementary Table 1), some of these elements are also briefly reported in the present report. All procedures were approved by the University of Arizona Institutional Review Board (Protocol 1904553365) and registered with ClinicalTrials.gov (NCT04796207). All participants provided written informed consent prior to enrollment.

Briefly, eligible participants were University of Arizona NCAA football players, age ≥18 years, cleared to participate in university athletics as determined by the team physician, and who volunteered to participate in the trial. Exclusionary criteria included (1) chronic use of anti-inflammatory medications (≥20 days); (2) antihypertensive medication use; (3) lipid-lowering medication use; active fish oil or omega-3 fatty acid supplementation; (4) self-reported consumption of more than two servings of fish per week; (5) injured or unable to participate in regularly scheduled conditioning or competitions; and (6) recent diagnosis of acute concussion. Where possible, exclusionary criteria were confirmed by sports medicine staff from student-athlete medical records. Furthermore, individuals sustaining a diagnosed concussion at any time during the trial were removed from participation. Enrolled participants were randomized to either the placebo or treatment group in a 1:1 ratio, counterbalanced by starter status based on the preceding season and estimated risk of RSHI by position (44). There were no changes in starter status over the course of the season for those participating in the trial. Finally, participants were included in the present analyses when both baseline and end of season neuroimaging sessions were available.

**Figure 1:**
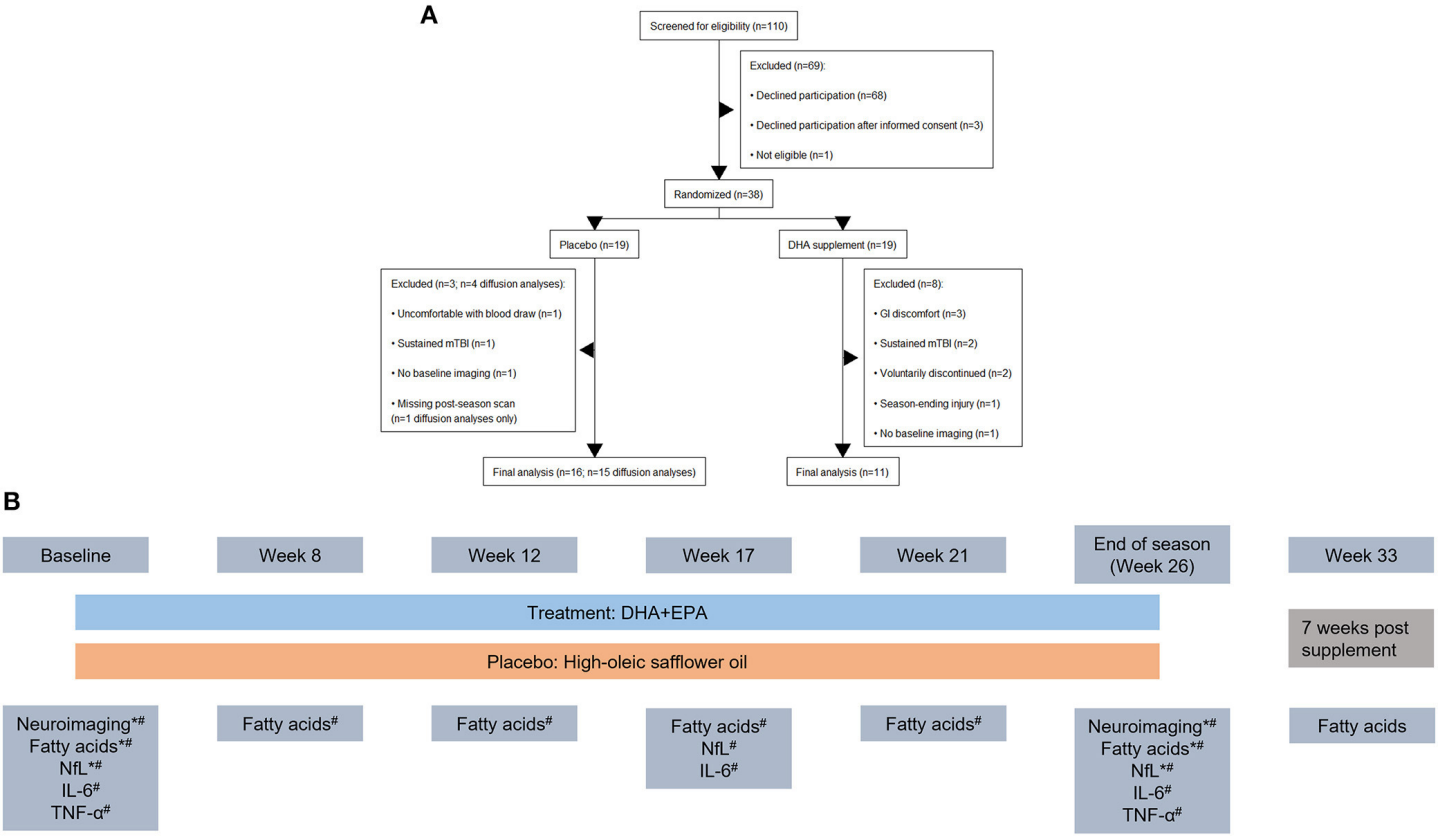
CONSORT diagram outlining participant flow through the study and study design. **(A)** Nineteen individuals were initially randomized to each arm of the study. After exclusions for study withdrawal and imaging completeness, a total of 27 individuals were analyzed for volumetric and functional connectivity outcomes and 26 individuals were analyzed for diffusion weighted imaging outcomes. **(B)** Study design schematic including time points for MRI and blood draws during the study. Outcomes reported in the present manuscript are denoted with a “*” and those reported in Mullins et al. (43) are denoted with a “#”. Blood-based biomarkers were collected together. Neuroimaging was collected in a 3-week (baseline) and 2-week (end of season) window around the blood draw.

A total of 38 football players were enrolled into the study. A total of 29 individuals completed the trial and had at least one session of neuroimaging scans. Two individuals were excluded from the neuroimaging analyses because they declined to complete the neuroimaging battery at the baseline assessment due to claustrophobia but did complete it at end of season. One individual was excluded from the diffusion weighted analyses due to a missing reverse phase-encoded field map at the end of season assessment. Thus a total of *n* = 27 individuals were included in the analyses of volumetric and functional connectivity findings (*n* = 11 in the treatment group) and *n* = 26 were included in the analyses of the diffusion data (*n* = 11 in the treatment group; Figure 1A). Participant characteristics are summarized in Table 1.

**Table 1:** Demographic characteristics of participants completing the trial and all imaging.

	**Placebo** ***n* = 16**	**DHA + EPA** ***n* = 11**	**Statistic (*p*-value)**
Age (mean ± SD)	21.2 ± 2.04	20.9 ± 0.70	*t*_19.7_ = 0.504 (0.62)
Height (in) (mean ± SD)	73.4 ± 2.17	72.2 ± 3.40	*t*_15.6_ = 1.06 (0.307)
Weight (lbs) (mean ± SD)	221.81 ± 42.14	208.91 ± 40.69	*T*_22.2_ = 0.798 (0.433)
Hispanic or Latino ethnicity (*n*)[%]	14 [87.5%]	11 [100%]	χ^2^ = 0.222 (0.638)
Race (*n*) [%]
Latino or Spanish origin	2 [12.5%]	0 [0%]	χ^2^ = 3.62 (0.305)
Black or African American	7 [43.75%]	5 [45.5%]	
White	5 [31.25%]	6 [54.5%]	
Multiple	2 [12.5%]	0 [0%]	
Starter (*n*) [%]	8 [50%]	6 [54.5%]	χ^2^ = 0 (1)
High risk position (*n*) [%]^a^	7 [43.75%]	5 [45.5%]	χ^2^ = 0 (1)

^a^High risk positions included offensive and defensive linemen, running backs, tight ends, and linebackers.

### Intervention

After baseline measures were obtained prior to the start of treatment, all participants took six 1.0g soft gel capsules each day, 5 days per week for approximately seven months (placebo: 713 mg of oleic acid and 130 mg of linoleic acid from high-oleic safflower oil per capsule; treatment: 407 mg of DHA and 170 mg of EPA as ethyl esters per capsule for a daily dose of 2.442 g/day DHA and 1.020 g/day EPA; Pharmavite, West Hills, CA). All capsules were color and size matched. Study staff delivered capsules to study participants at the football facility 5 days per week. Compliance was recorded by pill count with returned or unconsumed capsules counted and logged. Compliance was set at 80%. Complete details of the lipid profiles for the placebo and treatment capsules, overall study compliance, adverse events, and primary analyses of change in serum DHA and EPA levels are reported in Mullins et al. (43).

### Outcomes

As noted, all pre-registered analyses are reported in Mullins et al. (43). The neuroimaging outcomes reported here are exploratory. Based on previous literature demonstrating effects of RSHIs (20–24), as well as DHA (29–32, 40–42), we focused on indicators of white matter integrity in these analyses, primarily using diffusion kurtosis scalar maps. For thorough reporting and future study planning, we additionally analyzed and report changes in gray matter volume and functional connectivity. For the primary trial outcomes, whole blood was collected at the beginning of the study prior to treatment, monthly during the preseason and season up until the final game when treatment was terminated, and then 1 month after treatment ended to quantify retention (*n* = 7 time points; Figure 1B). Serum neurofilament light (NfL), a primary outcome in Mullins et al. (43), is the only whole blood biomarker reported here for correlation with diffusion outcomes.

Additionally, at baseline and end of season, all participants completed three self-reported measures of mental health and sleep quality. These included the Patient Health Questionnaire 9 (PHQ-9) as a measure of depression (45), the Insomnia Severity Index (ISI) as a measure of insomnia (46), and item SLQ-120 from the National Health and Nutrition Examination Survey as an indicator of daytime sleepiness. Scoring was conducted in accordance with established guidelines and higher values indicate greater depression (PHQ-9), insomnia (ISI), and daytime sleepiness (SLQ-120).

### Neuroimaging acquisition and methods

All participants underwent a whole-brain multimodal neuroimaging battery prior to the start of treatment during the offseason and again at the end of the season. All images were acquired on a 3T Siemens Skyra located at the University of Arizona. This imaging battery included two high resolution structural T1-weighted MPRAGEs (TE: 2.98 ms; TR: 2,300 ms; inversion time: 900 ms; flip angle: 9°; FoV: 256 mm × 240 mm; acquisition matrix: 256 mm × 240 mm; slice thickness: 1 mm; total slices: 176; voxel size: 1 mm^3^), a resting state functional magnetic resonance imaging scan (rs-fMRI; TE: 30 ms; TR: 3,000 ms; FoV: 240 mm × 240 mm; acquisition matrix: 80 × 80; slice thickness: 3 mm; total slices: 54; voxel size: 2.5 mm^3^; total duration: 9 min and 51 s), and a multi-band multi-shell diffusion weighted imaging sequence (TE: 119 ms; TR: 3,700 ms; FoV: 256 mm × 256 mm; acquisition matrix: 102 × 102; slice thickness: 2mm; total slices: 54; voxel size: 2.5mm^3^; total duration: 6 min and 43 s) with 30 diffusion weighted directions at *b* = 500 s/mm^2^, *b* = 1,000 s/mm^2^, and *b* = 2,000 s/mm^2^ as well as a single *b* = 0 s/mm^2^ image. A reverse phase-encoded *b* = 0 s/mm^2^ scan with the same parameters was also collected for distortion correction.

#### Diffusion weighted imaging pre-processing

All diffusion-weighted data were pre-processed using QSIPrep (v. 0.12.2) (47) which is based on Nipype 1.5.1 (48, 49) (RRID:SCR_002502). Details of the processing pipeline are described below and many internal operations of QSIPrep use Nilearn 0.6.2 (50) (RRID:SCR_001362) and Dipy (51). For more details of the pipeline, see the section corresponding to workflows in QSIPrep's documentation (https://qsiprep.readthedocs.io/en/latest/). The outputs of QSIPrep include a pre-processed T1-weighted anatomical image and a pre-preprocessed diffusion weighted image coregistered to the T1-weighted image. Following preprocessing, we applied two different analytic approaches.

##### DWI pre-processing: Anatomical data

A total of 4 T1-weighted (T1w) images were found within the input BIDS dataset. All of them were corrected for intensity non-uniformity (INU) using *N4BiasFieldCorrection* (52) (ANTs 2.3.1, RRID:SCR_004757). A T1w-reference map was computed after registration of 4 T1w images (after INU-correction) using *mri_robust_template* [FreeSurfer 6.0.1 (53)]. The T1w-reference was then skull-stripped using *antsBrainExtraction.sh* (ANTs 2.3.1), using OASIS as target template. Spatial normalization to the ICBM 152 Nonlinear Asymmetrical template version 2009c (54) (RRID:SCR_008796) was performed through nonlinear registration with antsRegistration (55) (ANTs 2.3.1, RRID:SCR_004757), using brain-extracted versions of both T1w volume and template. Brain tissue segmentation of cerebrospinal fluid (CSF), white-matter (WM) and gray-matter (GM) was performed on the brain-extracted T1w using FAST (56) (FSL 6.0.3:b862cdd5, RRID:SCR_002823).

##### DWI pre-processing: Diffusion data

Any images with a *b*-value <100 s/mm^2^ were treated as a *b* = 0 image. Marchenko Pastur Principal Component Analysis denoising as implemented in MRtrix3's *dwidenoise* (57) was applied with a 5-voxel window. After denoising, Gibbs unringing was performed using MRtrix3's *mrdegibbs* (58) and B1 field inhomogeneity was corrected using *dwibiascorrect* from MRtrix3 with the N4 algorithm (52). After B1 bias correction, the mean intensity of the Diffusion weighted imaging (DWI) series was adjusted so all the mean intensity of the *b* = 0 images matched across each separate DWI scanning sequence.

FSL's (version 6.0.3:b862cdd5) *eddy* was used for head motion correction and eddy current correction (59). Eddy was configured with a q-space smoothing factor of 10, a total of five iterations, and 1,000 voxels used to estimate hyperparameters. A linear first level model and a linear second level model were used to characterize Eddy current-related spatial distortion. *q*-space coordinates were forcefully assigned to shells. Field offset was attempted to be separated from subject movement. Shells were aligned post-eddy. Eddy's outlier replacement was run (59). Data were grouped by slice, only including values from slices determined to contain at least 250 intracerebral voxels. Groups deviating by more than four standard deviations from the prediction had their data replaced with imputed values (maximum range: 0.67%−2.8% of 4,914 total slices per subject per session). Data was collected with reversed phase-encode blips, resulting in pairs of images with distortions going in opposite directions. Here, *b* = 0 reference images with reversed phase encoding directions were used along with an equal number of *b* = 0 images extracted from the DWI scans. From these pairs the susceptibility-induced off-resonance field was estimated using a method similar to that described in Andersson et al. (60). The fieldmaps were ultimately incorporated into the Eddy current and head motion correction interpolation. Final interpolation was performed using the *jac* method.

Several confounding time-series were calculated based on the preprocessed DWI: framewise displacement (FD) using the implementation in Nipype [following the definitions by Power et al. (61)]. The head-motion estimates calculated in the correction step were also placed within the corresponding confounds file. Slicewise cross correlation was also calculated. The DWI time-series were resampled to ACPC, generating a preprocessed DWI run in anterior commissure-posterior commissure space with 1mm isotropic voxels.

##### Tract-based spatial statistics

Diffusion tensor and kurtosis parameters were computed using DIPY (v. 1.3.0) (51). Extracted scalar maps included fractional anisotropy (FA), mean (MD), axial (AD), and radial diffusivity (RD), axial (AK) and radial kurtosis (RK), kurtosis fractional anisotropy (KFA), and the mean of the kurtosis tensor (MKT). Scalar data were processed using a modified version of FSL's *tbss* pipeline (62, 63) (FSL v. 6.0.3; RRID:SCR_002823) for voxel-wise analyses. Specifically, the FA maps were eroded using the standard *tbss_1_preproc* function. Next, a study-specific nonlinear template was created from the pre-processed T1-weighted images from QSIPrep using *antsMultivariateTemplateConstruction2* (ANTs 2.2.0) (64). Individual FA maps were then warped to the study-specific template from the computed warps using *antsApplyTransforms*. FA images were concatenated and masked as usual using commands from *tbss_3_postreg*. The mean FA map was created and skeletonised within that mask and the individual FA maps were projected onto the mean skeleton and masked using a threshold of 0.2 for analyses. The scalar maps for MD, AD, RD, AK, RK, KFA, and MKT were warped to the template in similar fashion and projected onto the mean skeleton. Difference maps per subject (end of season – baseline) were computed on the skeletonised scalar maps for analyses.

##### Whole-brain deterministic tractography

Preprocessed diffusion weighted data were also processed using DSI Studio (2021-03-11 release; http://dsi-studio.labsolver.org/). Using the QSIPrep preprocessed images, the accuracy of the b-table was checked by an automatic quality control routine (65) and neighboring DWI correlation was computed as a quality control measure. The diffusion data were reconstructed in Montreal Neurological Institute (MNI) space using q-space diffeomorphic reconstruction (66) to obtain the spin distribution function (67) with a diffusion sampling length of 1.25. Quantitative anisotropy was extracted as the local connectome fingerprint (68) and used in the connectometry analyses.

#### Volumetric pre-processing

All volumetric analyses were conducted in the CAT12 toolbox (v 12.8.1; http://www.neuro.uni-jena.de/cat/) for SPM12 (r7771; https://www.fil.ion.ucl.ac.uk/spm/software/spm12/) and executed in MATLAB 2020a. For each session, one of the T1-weighted images was selected among the two available based on the highest signal-to-noise ratio and visual inspection of imaging artifacts. These images were pre-processed using the CAT12.8.1 longitudinal segmentation pipeline with an output resolution of 1mm^3^. All other default parameters were used and quality checked. This pre-processing stream resulted in bias-corrected, segmented, and spatially normalized images in MNI152NLin2009cAsym space (54). Regional gray matter volume was computed in CAT12 for an atlas combining the Schaefer 400x17 network cortical ROIs (69), 36 subcortical ROIs from the Brainnetome atlas (70), and 37 cerebellar ROIs (71). This is the same atlas used for functional connectivity and regional homogeneity analyses described in section “Functional connectivity.”

#### Resting state FMRI (rs-FMRI) pre-processing

Resting-state fMRI pre-processing was conducted in fMRIPrep (v 20.1.1) (RRID:SCR_016216) (72, 73), which is based on Nipype 1.5.0 (RRID:SCR_002502) (48, 49). Many internal operations of fMRIPrep use Nilearn 0.6.2 (50) (RRID:SCR_001362), mostly within the functional processing workflow. For more details of the pipeline, see the section corresponding to workflows in fMRIPrep's documentation (https://fmriprep.org/en/20.1.1/). The descriptions of the pre-processing steps in fMRIPrep are provided by the creators of the software under a CC0 license and reproduced here without changes.

##### rs-fMRI pre-processing: Anatomical data

A total of 4 T1-weighted (T1w) images were found within the input BIDS dataset. All of them were corrected for intensity non-uniformity (INU) with *N4BiasFieldCorrection* (52), distributed with ANTs 2.2.0 (55) (RRID:SCR_004757). The T1w-reference was then skull-stripped with a Nipype implementation of the *antsBrainExtraction.sh* workflow (from ANTs), using OASIS30ANTs as the target template. Brain tissue segmentation of cerebrospinal fluid (CSF), white-matter (WM) and gray-matter (GM) was performed on the brain-extracted T1w using *fast* (56) (FSL 5.0.9, RRID:SCR_002823). A T1w-reference map was computed after registration of three T1w images (after INU-correction) using *mri_robust_template* (53) (FreeSurfer 6.0.1). Brain surfaces were reconstructed using *recon-all* (74) (FreeSurfer 6.0.1, RRID:SCR_001847), and the brain mask estimated previously was refined with a custom variation of the method to reconcile ANTs-derived and FreeSurfer-derived segmentations of the cortical gray-matter of Mindboggle (75) (RRID:SCR_002438). Volume-based spatial normalization to two standard spaces (MNI152NLin2009cAsym, MNI152NLin6Asym) was performed through nonlinear registration with *antsRegistration* (ANTs 2.2.0), using brain-extracted versions of both T1w reference and the T1w template. The following templates were selected for spatial normalization: *ICBM 152 Nonlinear Asymmetrical template version 2009c* (54) [RRID:SCR_008796; TemplateFlow ID: MNI152NLin2009cAsym], *FSL's MNI ICBM 152 non-linear 6th Generation Asymmetric Average Brain Stereotaxic Registration Model* (76) [RRID:SCR_002823; TemplateFlow ID: MNI152NLin6Asym].

##### rs-fMRI pre-processing: Functional data

For each of the two BOLD runs found per subject (across all sessions), the following preprocessing was performed. First, a reference volume and its skull-stripped version were generated using a custom methodology of fMRIPrep. Head-motion parameters with respect to the BOLD reference (transformation matrices, and six corresponding rotation and translation parameters) are estimated before any spatiotemporal filtering using *mcflirt* (77) (FSL 5.0.9). BOLD runs were slice-time corrected using 3dTshift from AFNI 20160207 (78) (RRID:SCR_005927). Susceptibility distortion correction (SDC) was omitted. The BOLD reference was then co-registered to the T1w reference using *bbregister* (FreeSurfer) which implements boundary-based registration (79). Co-registration was configured with six degrees of freedom. The BOLD time-series (including slice-timing correction when applied) were resampled onto their original, native space by applying the transforms to correct for head-motion. These resampled BOLD time-series will be referred to as *preprocessed BOLD in original space*, or just *preprocessed BOLD*.

The BOLD time-series were resampled into several standard spaces, correspondingly generating the following spatially-normalized, preprocessed BOLD runs: MNI152NLin2009cAsym, MNI152NLin6Asym. First, a reference volume and its skull-stripped version were generated using a custom methodology of fMRIPrep. Several confounding time-series were calculated based on the preprocessed BOLD: framewise displacement (FD), DVARS and three region-wise global signals. FD was computed using two formulations following Power (absolute sum of relative motions) (61) and Jenkinson (relative root mean square displacement between affines) (77). FD and DVARS are calculated for each functional run, both using their implementations in Nipype [following the definitions by Power et al. (61)]. The three global signals are extracted within the CSF, the WM, and the whole-brain masks. Additionally, a set of physiological regressors were extracted to allow for component-based noise correction (CompCor) (80). Principal components are estimated after high-pass filtering the preprocessed BOLD time-series (using a discrete cosine filter with 128s cut-off) for the two CompCor variants: temporal (*tCompCor*) and anatomical (*aCompCor*). *tCompCor* components are then calculated from the top 5% variable voxels within a mask covering the subcortical regions. This subcortical mask is obtained by heavily eroding the brain mask, which ensures it does not include cortical GM regions. For *aCompCor*, components are calculated within the intersection of the aforementioned mask and the union of CSF and WM masks calculated in T1w space, after their projection to the native space of each functional run (using the inverse BOLD-to-T1w transformation). Components are also calculated separately within the WM and CSF masks. For each CompCor decomposition, the *k* components with the largest singular values are retained, such that the retained components' time series are sufficient to explain 50 percent of variance across the nuisance mask (CSF, WM, combined, or temporal). The remaining components are dropped from consideration.

The head-motion estimates calculated in the correction step were also placed within the corresponding confounds file. The confound time series derived from head motion estimates and global signals were expanded with the inclusion of temporal derivatives and quadratic terms for each (81). Frames that exceeded a threshold of 0.5 mm FD or 1.5 standardized DVARS were annotated as motion outliers. All resamplings can be performed with a single interpolation step by composing all the pertinent transformations (i.e. head-motion transform matrices, susceptibility distortion correction when available, and co-registrations to anatomical and output spaces). Gridded (volumetric) resamplings were performed using *antsApplyTransforms* (ANTs), configured with Lanczos interpolation to minimize the smoothing effects of other kernels (82). Non-gridded (surface) resamplings were performed using *mri_vol2surf* (FreeSurfer).

##### Functional connectivity

Post-processing, functional connectivity, and regional homogeneity estimation from the *preprocessed BOLD time series* (in MNI152NLin2009cAsym space) was accomplished for each subject and session using the eXtensible Connectivity Pipeline (XCP Engine, v 1.2.3; https://xcpengine.readthedocs.io/). Confound regressors were collected from fMRIPrep and included the mean global, cerebrospinal fluid, and white matter signals, framewise motion (*x*-, *y*-, and *z*- axis translation and rotation) as well as the derivatives and quadratic expansions of these terms (36 parameter confound regressors) (83). Processing steps included demeaning, detrending, and temporal filtering (0.01–0.08 Hz Butterworth filter) of the time series and regressors, as well as despiking of the BOLD time-series using *3dDespike* from AFNI (78) (RRID:SCR_005927) followed by regression using the 36 parameters. The residual BOLD time-series following regression was the averaged in each of the 473 ROIs (69–71) used in estimating the gray matter volume. However, due to varying levels of field of view coverage for the cerebellum during the resting state imaging, the 37 cerebellar ROIs were ultimately excluded. Functional connectivity was then computed as the Pearson *r* correlation between each ROI, yielding a 436 × 436 functional connectivity matrix per subject per session (400 cortical ROIs, 36 subcortical ROIs). These connectivity matrices were subsequently Fisher *r-to-z* transformed to improve the normality of the distribution of the correlation coefficients. Additional quality metrics provided by XCP Engine included mean root mean square (RMS) motion estimates and node coverage.

#### Neurofilament-light blood-based biomarker

Whole blood was collected (1) prior to non-contact conditioning (baseline); (2) at weeks 8, 12, 17, 21, 26, and 33 (Figure 1B). Blood draws during the regular season [Weeks 12, 17, 21, and 26 (end of season)] were done on Tuesday or Wednesday following game day. Week 33 was 7 weeks after the end of treatment. Twelve-hour fasted blood samples (22 ml whole blood) were collected via venipuncture into three vacutainer tubes [1 × 4.5 ml tri-sodium citrate, 1 × 7.5 ml powdered glass clot activator, 1 × 10 ml ethylene diamine tetra-acetic acid (EDTA)]. The tri-sodium citrate tube was stored on ice, packaged, and sent for analysis. Samples in the EDTA tube were centrifuged within 2 min of collection (1,714 g for 15 min). Samples in the clot activator tube were centrifuged 30 min post-collect (1,714 g for 15 min). Aliquots of red blood cells and plasma (EDTA tube) and serum (clot activator tube) were stored at−80C until analyzed. Serum neurofilament light (NfL) was quantified at baseline, Week 17, and Week 26 from serum using the NF-Light Simoa Assay Advantage Kit (Quanterix Corp, Billerica, MA). Other blood-based biomarkers included plasma fatty acids, interleukin-6 and tumor necrosis factor-alpha, all of which are thoroughly described in Mulllins et al. (43). For treatment effects in the present analyses, DHA and EPA data are also reported here.

### Statistical methods

Of the 27 individuals who completed pre and post season imaging and trial data, one individual was excluded from the diffusion weighted analyses due to a missing reverse phase-encoded field map at the postseason assessment. Thus a total of *n* = 27 individuals were included in the analyses of volumetric and functional connectivity findings (*n* = 11 in the treatment group) and *n* = 26 were included in the analyses of the diffusion data (*n* = 11 in the treatment group; Figure 1). Participant characteristics are summarized in Table 1.

#### Demographic, serum, and behavioral outcomes

Complete demographic characteristics and outcomes for all measured blood-based outcomes are reported in Mullins et al. (43). Here we reported specifically the outcomes for NfL, DHA, and EPA in the subset of individuals included in the imaging analyses. Demographic characteristics were assessed using unpaired t-tests for age, height, and weight. Self-report race and ethnicity, as well as starter status and RSHI risk position status, were compared between groups using chi-squared. Changes in serum measures (NfL, DHA, and EPA) and behavioral measures (PHQ-9, ISI, SLQ-120) were assessed using a modified baseline-adjusted ANCOVA model. Specifically, end of season values were residualized on baseline values using a linear model in R version 4.2 (84). Treatment-related between-group differences were then computed in Python (3.7.6) using DABEST (85). DABEST computes measures of effect size and 95% confidence intervals using bias-corrected accelerated bootstrapping. Effect sizes are reported as Hedges *g*. Confidence intervals were computed using 20,000 bootstrap resamples, and *p*-values were computed using 5,000 permutations.

#### Tract-based spatial statistics

The primary white matter outcome of interest was the between group difference in white matter integrity change, quantified *via* the diffusion tensor and kurtosis scalar maps as well as connectometry. A two-sample *T*-test was conducted on each of the six scalar difference maps (FA, MD, AD, RD, AK, RK, KFA, and MKT) using *randomise* in FSL. Threshold-free cluster enhancement (TFCE) with 5,000 permutations was used to identify statistically significant differences in group-wise changes in scalar metrics between baseline and end of season. All analyses were conducted at family-wise error (FWE) corrected *p* < 0.05. For analyses with statistically significant findings, a binary mask of significant voxels was created and the mean value across those voxels was extracted for each person. We additionally applied the same approach using a one-sample permutation *t*-test to identify statistically significant increases and decreases in these six scalars over the course of the season irrespective group.

Following the treatment-related analyses, we further investigated the association between baseline-to-end of season changes in serum NfL and measures of change in white matter integrity. For the diffusion tensor and kurtosis scalars (FA, MD, AD, RD, AK, RK, FKA, MKT), voxel-wise associations between the skeletonised difference maps and change in NfL (end of season – baseline) were assessed via *randomise* using 5,000 permutations and reported for FWE-corrected *p* < 0.05.

#### Deterministic tractography

For whole-brain tractography, diffusion MRI connectometry (86) was used to derive correlational tractography demonstrating a longitudinal change in quantitative anisotropy (QA; postseason – baseline) associated with treatment group assignment (1 = DHA, −1 = Placebo). A nonparametric Spearman correlation was used to derive the correlation. A total of 26 subjects were included in the analysis. A *T*-score threshold of 2.5 was assigned and tracked using a deterministic fiber tracking algorithm (87) to obtain correlational tractography and the QA values were normalized for analyses (range 0–1 within subject). The tracks were filtered by topology-informed pruning (88) with four iteration(s). A length threshold of 20 mm was used to select tracks. To estimate the false discovery rate (FDR), a total of 4,000 randomized permutations were applied to the treatment group label to obtain the null distribution of the track length. Statistically significant correlations were defined at an FDR corrected *p* < 0.05.

For connectometry analyses of the association between change in QA and change in NfL, a T-score threshold of 3.0 was assigned and tracked using a deterministic fiber tracking algorithm (87) to obtain correlational tractography. A nonparametric Spearman correlation was used to derive the correlation. A total of 26 subjects were included in the analysis. The tracks were filtered by topology-informed pruning (88) with 4 iteration(s) and the QA values were normalized for analyses (range 0–1 within subject). A length threshold of 20mm was used to select tracks. To estimate the false discovery rate, a total of 4,000 randomized permutations were applied to the NfL change data to obtain the null distribution of the track length. Significant correlations were defined at FDR corrected *p* < 0.05.

#### Volumetric changes

Changes in gray matter volume in the 473 ROIs described in section “Neuroimaging acquisition and methods” were modeled using a modified baseline-adjusted ANCOVA approach. First, baseline and end of season gray matter volume for each ROI was residualized for total brain volume (TBV; computed as the sum of gray and white matter volume). While TBV is expected to be stable over this time period, small differences in registration and tissue contrast may exist and therefore each session was residualized separately. Second, end of season TBV-adjusted gray matter volume was residualized on baseline TBV-adjusted gray matter volume. Treatment-related between-group differences were then computed per ROI with DABEST (85) using 20,000 bootstrap resamples for the effect size 95% confidence interval and 5,000 permutations for the empirical *p*-value. Additionally, overall baseline to end of season changes were analyzed using the TBV-adjusted values and a paired data approach in DABEST, similarly using 20,000 bootstraps and 5,000 permutations. Reported outcomes exclude ROIs with a confidence interval including 0 and are further thresholded at an FDR corrected *p* < 0.05 level.

#### Inter-regional functional connectivity

Atlas-based resting-state analyses were conducted on the processed outputs from XCP Engine in R (v 4.0.0). First, all ROI-to-ROI correlations (hereafter: edges) were Fisher *r-*to-*z* transformed within an atlas including the Schaefer 400 ROI 17-network atlas (69) and the subcortical ROIs from the Brainnetome atlas (70). Then, two outcomes of interest were investigated. First, group-wise differences in whole-brain edge-wise functional connectivity were modeled as a preseason-adjusted ANCOVA. Specifically, end of season connectivity values were residualized on baseline connectivity for each edge. Treatment-related between-group differences were then computed in Python (3.7.6) using DABEST (85). DABEST computes measures of effect size and 95% confidence intervals using bias-corrected accelerated bootstrapping. All effect sizes here are reported as Hedges *g*. Confidence intervals were computed using 20,000 bootstrap resamples, and *p*-values were computed using 5,000 permutations. To minimize false positives, the list of edges was initially thresholded to exclude confidence intervals including 0 and then thresholded at an FDR-corrected *p* < 0.01. This approach has previously been used to identify edge-wise treatment effects on functional connectivity in other populations (89).

Second, between-group differences in changes in network-wise functional connectivity were investigated. Each of the ROIs was assigned to one of nine networks based on the Yeo 17-network parcellation [using overarching network names when multiple sub-networks exist; e.g., the default mode A, B, and C networks were labeled as DMN] for the cortical ROIs and a sub-cortical network for the Brainnetome subcortical ROIs. For each of the networks, the average weighted mean within-network and cross-network functional connectivity was computed as previously defined (90). Group-wise differences in network connectivity changes were computed using a baseline-adjusted ANCOVA and DABEST. Statistically significant differences were identified using the same thresholding procedure.

#### Intra-regional functional connectivity

ReHo was investigated in a similar manner to the edge-wise functional connectivity. For each ROI in the same atlas that has been described, a baseline-adjusted ANCOVA followed by bootstrapped effect size calculation in DABEST was used. Statistically significant ROIs were identified using initial thresholding on confidence intervals followed by FDR corrected *p* < 0.05.

## Results

A total of 27 (*n* = 11 in the Treatment group) completed baseline and end of season neuroimaging as well as all other study-related protocols. The groups were well-matched in all demographics (Table 1) and counterbalanced between starters/non-starters as well as low/high risk positions, even with dropouts after allocation. Baseline NfL values did not differ between groups (Table 2); however DHA [*g* = −1.00, 95% CI: (−1.52, −0.27), *p* = 0.008] and EPA [*g* = −0.77, 95% CI: (−1.31, −0.01), *p* = 0.032] percentage were both greater in the placebo group than the treatment group at baseline. After controlling for baseline values, NfL levels at the end of the season did not differ between treatment and placebo groups. DHA levels were significantly increased in the treatment group and EPA increased but did not meet a statistically significant *p* < 0.05 level (Table 2).

**Table 2:** Serum and behavioral biomarker changes from baseline to end of season in participants completing the trial and all imaging.

	**Placebo** ***n*** = **16**		**DHA** + **EPA** ***n*** = **11**		**Hedges *g* [95% CI]^a^**	***p*-Value^b^**
	**Baseline**	**End of season**	**Baseline**	**End of Season**		
NfL (pg/ml)	4.86 ± 1.20	6.38 ± 1.31	5.06 ± 2.07	7.63 ± 3.91	0.46 [−0.48, 1.25]	0.2744
DHA (%)	1.70 ± 0.57	1.45 ± 0.37	1.22 ± 0.26	2.10 ± 0.66	1.24 [0.45, 1.93]	0.0018
EPA (%)	0.50 ± 0.23	0.48 ± 0.22	0.35 ± 0.11	0.72 ± 0.53	0.72 [−0.05, 1.42]	0.0616
PHQ-9	1.25 ± 1.61	1.94 ± 2.26	1.30 ± 1.64	1.82 ± 2.14	−0.02 [−0.74, 0.88]	0.9518
ISI	2.56 ± 2.45	3.06 ± 4.09	5.55 ± 4.97	5.00 ± 5.37	−0.30 [−1.05, 0.62]	0.4536
SLQ-120	1.62 ± 0.86	2.12 ± 0.86	1.18 ± 0.98	1.09 ± 1.14	−0.84 [−1.63, 0.04]	0.0286

^a^Reported effect size is for the between-groups differences in baseline adjusted end of season biomarker values. Confidence intervals were computed using 20,000 bootstrap resamples. Positive values indicate Treatment > Placebo.

^b^p-Values are empirical p-Values for the between-groups difference in baseline-adjusted end of season biomarker values. p-Value were computed using 5,000 permutations of the group label.

NfL, neurofilament light; DHA, docosahexaenoic acid; EPA, eicosapentaenoic acid; PHQ-9, Patient Health Questionnaire 9; ISI, Insomnia Severity Index; SLQ-120, National Health and Nutrition Examination Survey Sleep Disorders Questionnaire item 120.

At baseline, the treatment group reported greater insomnia severity [*g* = 0.79, 95% CI: (−0.09, 1.61), *p* = 0.047] than the placebo group, but not greater depression or daytime sleepiness. After adjusting for baseline values, the treatment group reported significantly less daytime sleepiness, but not depression or insomnia (Table 2).

### Axonal damage from football participation is not ameliorated by DHA + EPA supplementation

**Figure 2:**
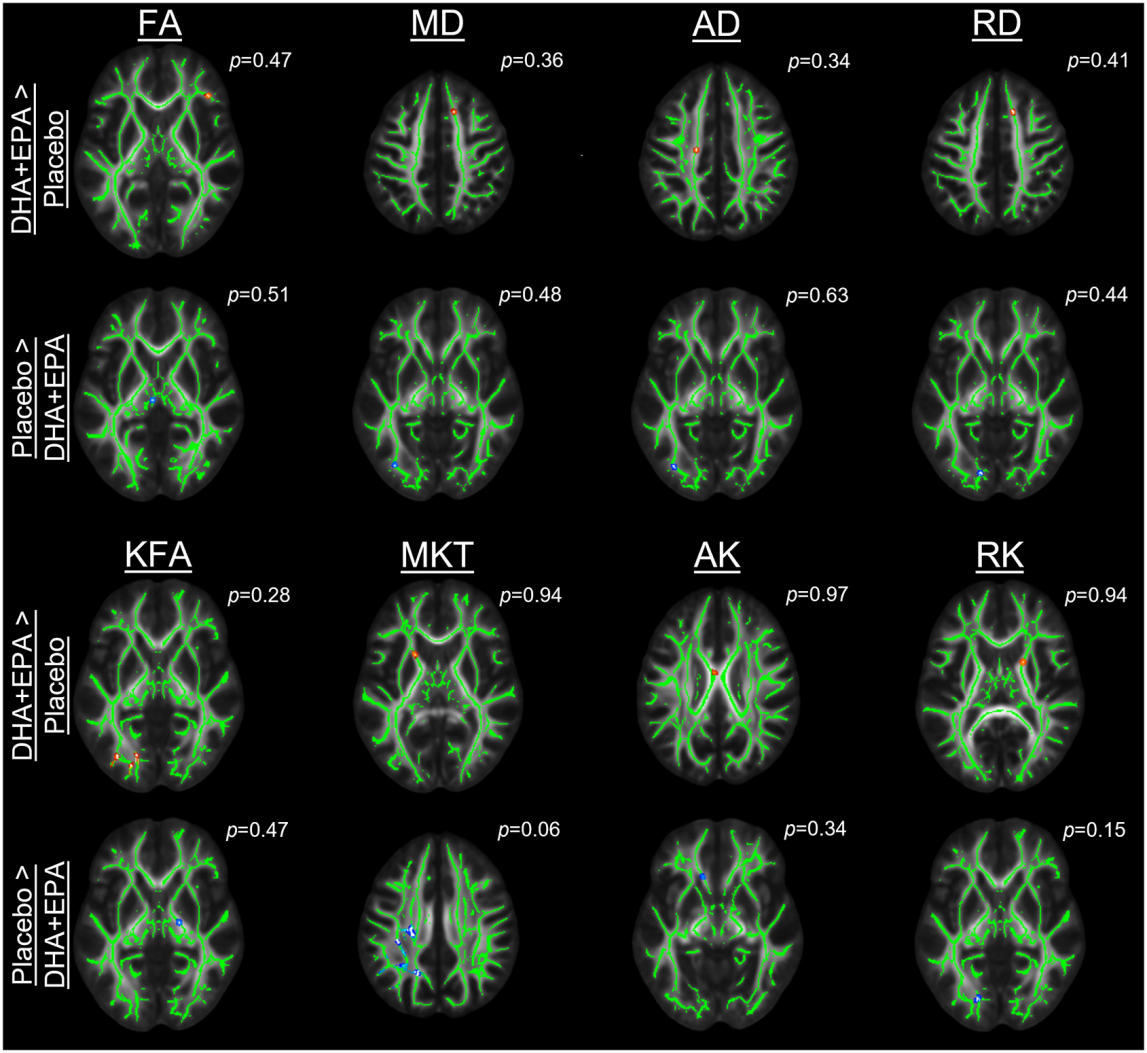
Group-wise differences in diffusion tensor and kurtosis metrics following treatment. Changes in diffusion tensor and kurtosis metrics were evaluated between groups using tract-based spatial statistics. No statistically significant clusters were observed for diffusion tensor metrics (FA, MD, AD, RD). For diffusion kurtosis metrics, a cluster of voxels (*n* = 1,704) was identified where the placebo groups demonstrated a greater increase in the mean of the kurtosis tensor (MKT) than the DHA + EPA group. Other diffusion kurtosis metrics (KFA, AK, RK) revealed no differences. For each analysis, results are overlaid on the template brain with the skeletonized white matter voxels in green. Statistical images are presented for the smallest observed family-wise error corrected *p*-value for each contrast (DHA + EPA > Placebo: top contrast, red-yellow voxels; Placebo > DHA: bottom contrast, blue-light blue voxels).

Outcomes of these analyses indicated no statistically significantly different changes in white matter metrics between placebo and DHA+EPA groups in any of the tensor-based voxel-wise analyses (Figure 2). However, for the mean of the kurtosis tensor (MKT), a cluster of voxels (*n* = 1,704; FWE corrected *p* ≤ 0.06) was identified in the superior and posterior corona radiata as well as superior longitudinal fasciculus where MKT increased in the placebo group (mean change = 0.049 ± 0.017) and decreased in the DHA+EPA group (mean change = −0.048 ± 0.059), though this cluster did not meet the *a priori* threshold for statistical significance.

To assess whether the lack of between-group differences was due to a lack of change over time, we conducted one-sample t-tests on the change maps. Analyses of changes from baseline to end of season revealed decreased axial diffusivity in three clusters of voxels (*n* = 1,182, FWE corrected *p* = 0.046; *n* = 1,795, FWE corrected *p* = 0.048; *n* = 3,768, FWE corrected *p* = 0.044). These voxels were primarily located in the left hemisphere's superior longitudinal fasciculus, superior corona radiata, posterior limb of the internal capsule, and body of the corpus callosum. Likewise a cluster was identified (*n* = 4,084 voxels; FWE corrected *p* < 0.055) in many of the same regions with decreased mean diffusivity, but this failed to reach the *a priori* level of statistical significance.

### Preserved structural connectivity in ascending fiber tracts is associated with DHA + EPA supplementation

**Figure 3:**
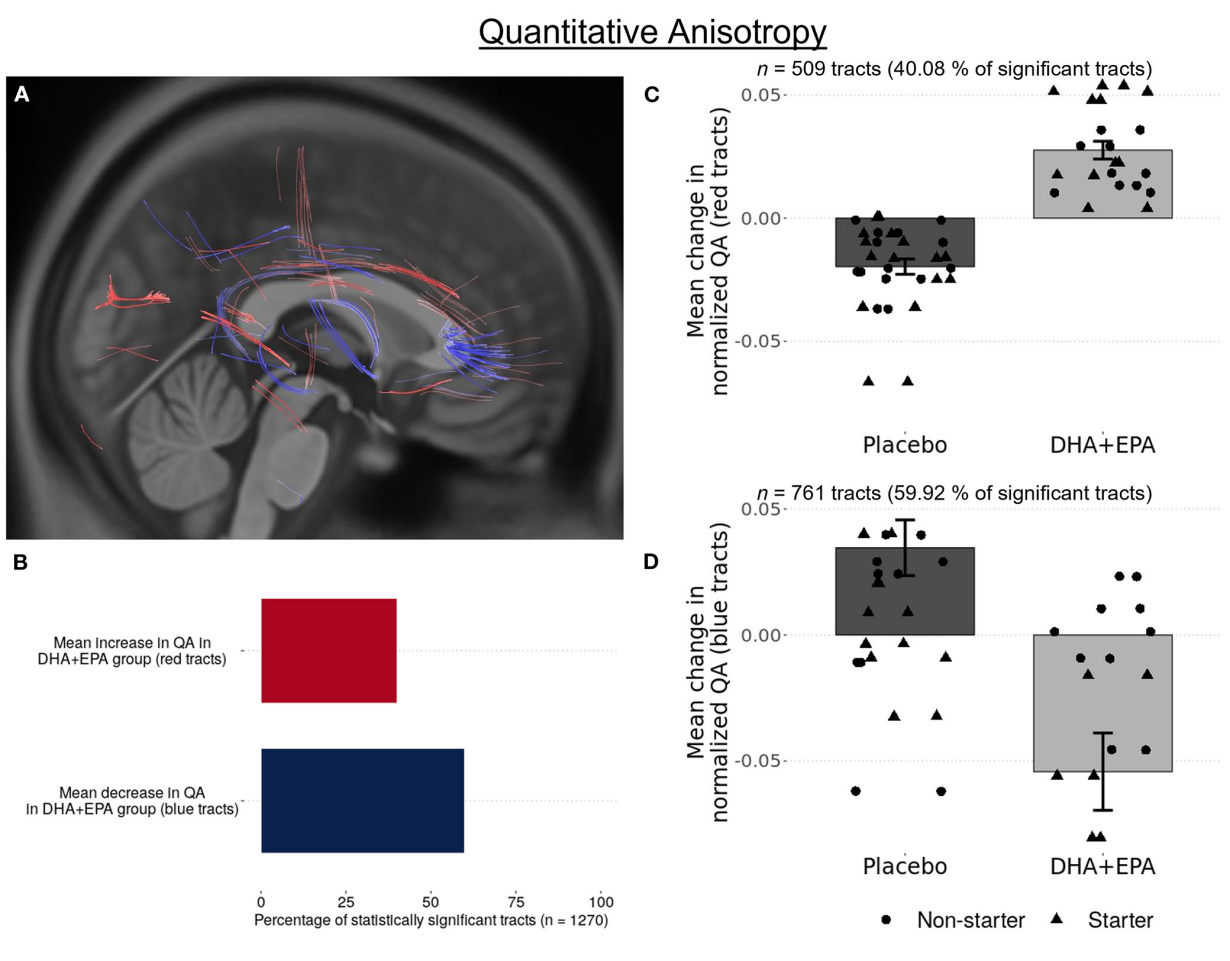
Group-wise differences in white matter structural connectivity following treatment. Quantitative anisotropy (QA) was used to create local connectome fingerprints to track white matter changes. A total 1,270 survived multiple comparisons correction of (FDR corrected *p* < 0.003). 40.08% of these tracts showed an increase in QA for participants receiving DHA + EPA and a decrease in QA for participants receiving placebo (**A** red-colored tracts; **B,C**). 59.91% of the identified tracts exhibited increased QA in participants receiving placebo and a decrease in QA for those receiving DHA + EPA (**A** blue-colored tracts; **B,D**).

Using deterministic tractography, a total of 509 tracts were identified with greater QA change in the DHA + EPA group compared to the placebo group, primarily in the left anterior and superior corticostriatal tracts as well as the right acoustic radiation (FDR corrected *p* = 0.003, Figure 3A red tracts, 3B, C). In addition, 761 white matter tracts exhibited a statistically significant increase change in QA in the placebo group whereas QA in the DHA + EPA group decreased from baseline to end of season (FDR corrected *p* = 0.003; Figure 3A blue tracts 3B, D). These tracts with decreased QA in the DHA + EPA group were primarily in the genu of the corpus callosum and the right anterior corticostriatal tract.

### Gray matter volume decreases over the course of a single football season

**Figure 4:**
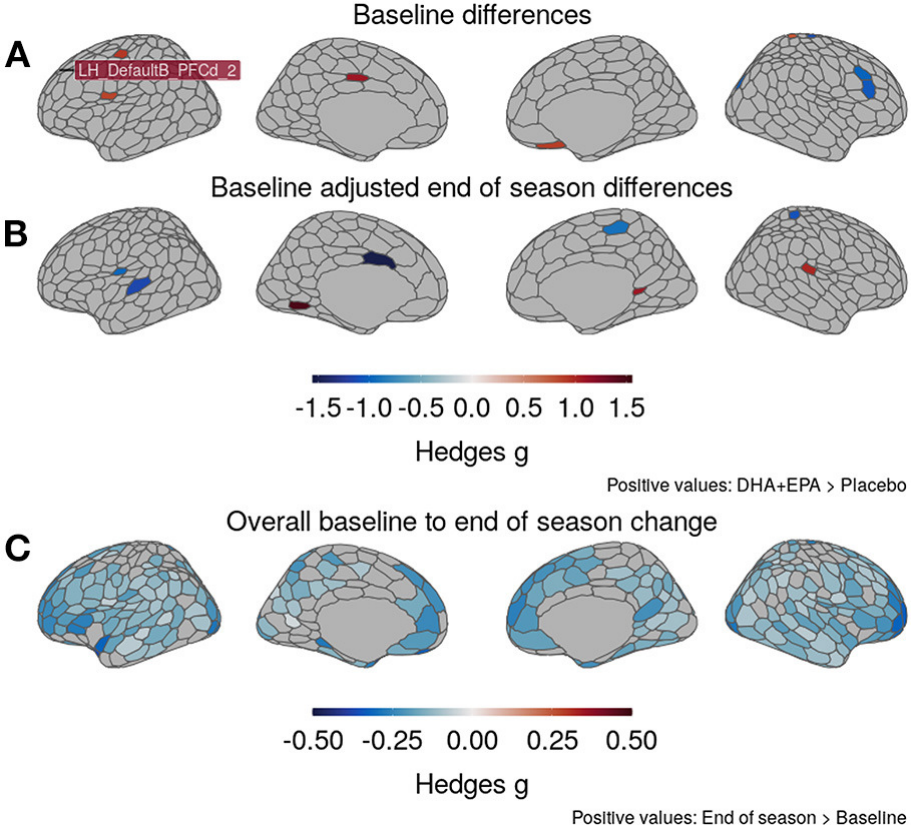
Changes in gray matter volume from baseline to end of season. **(A)** DHA + EPA and Placebo groups exhibited moderate to large, statistically significant differences in gray matter volume in 10 regions prior to treatment. **(B)** Baseline-adjusted models revealed eight regions with moderate-to-large, significant between-group differences at the end of the season that differed from those at baseline. **(C)** Irrespective of groups, moderately large, statistically significant decreases in gray matter volume were observed in 280/473 regions, including those identified to differ between groups. No regions exhibited increased volume. Specific region names for those identified in **(A)** and **(B)** reported in Supplementary Table 2.

At baseline (Figure 4A), greater volume was observed in the DHA + EPA group in six ROIs distributed primarily in the right hemisphere, while greater volume was noted in four ROIs for the placebo group. After controlling for baseline volumes, the treatment group had greater volume in three ROIs compared to the placebo group while the placebo group had greater volume in five ROIs (Figure 4B). A complete list of region names and effect sizes for baseline and baseline-adjusted end of season differences is included in Supplementary Table 2. Given the small number of between-group differences, we further assessed the main effect of time on gray matter volume. Small to moderate decreases in gray matter volume were observed in 280/473 ROIs, including ROIs, distributed across both hemispheres (Figure 4C), the right nucleus accumbens and right ventromedial putamen, and 14 cerebellar ROIs.

### DHA + EPA supplementation preserves default mode – frontoparietal control cross-network functional connectivity

**Figure 5:**
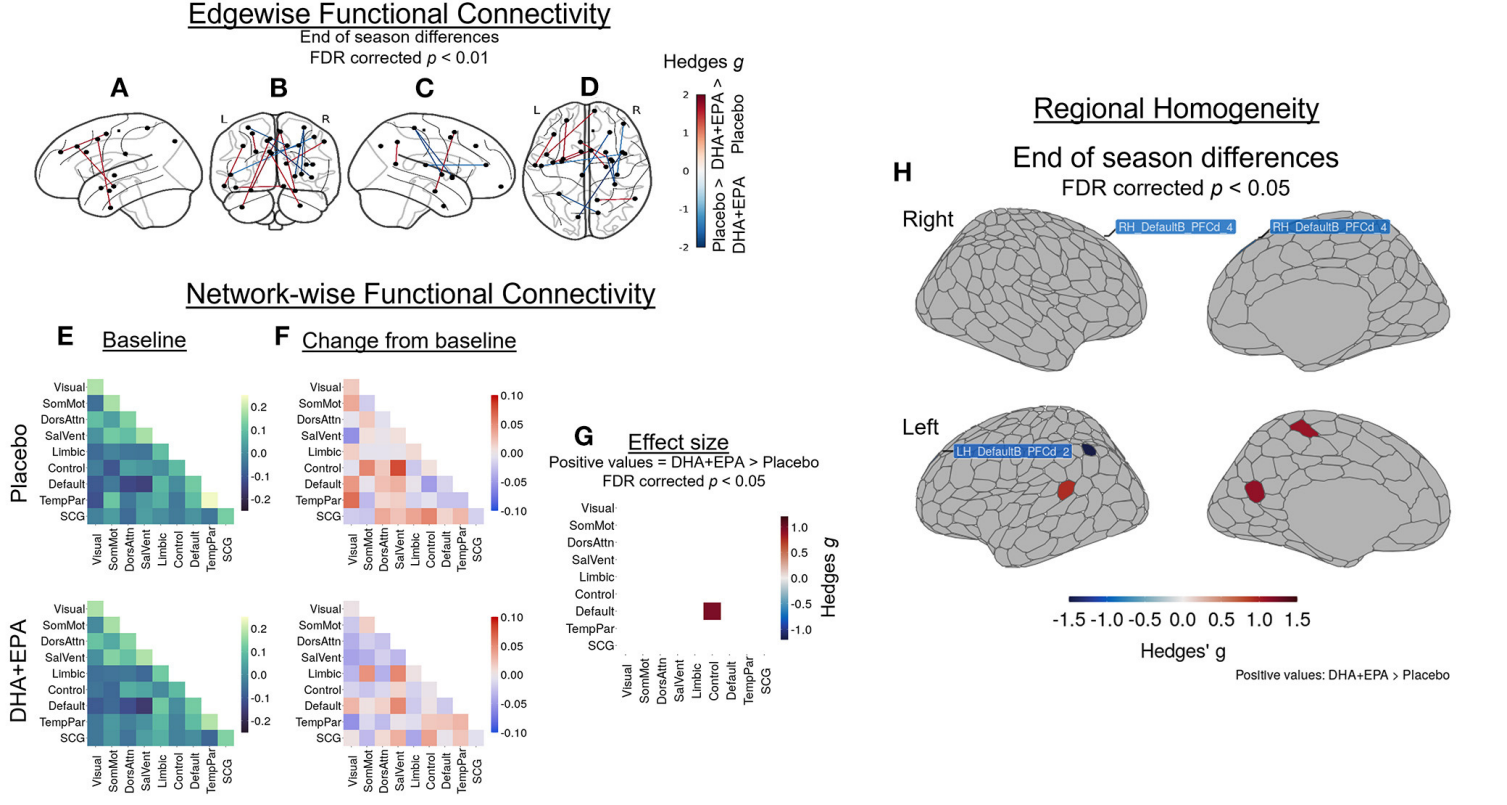
Group-wise differences in changes in inter- and intra-regional functional connectivity following treatment. Changes in functional connectivity were evaluated using baseline-adjusted ANCOVA models at an inter-regional (“edgewise”) **(A–D)**, network **(E–G)**, and local (regional homoegeneity) level. Findings are reported as Hedges *g* effect sizes. A total of 14 edges exhibited large between-group differences (|*g*| > 1.48, FDR corrected *p* < 0.01) and DHA + EPA supplementation was associated with increased connectivity in nine edges, primarily linking salience/ventral attention, limbic, and subcortical regions. These edgewise connections are overlaid on the left hemisphere surface **(A)**, a coronal view **(B)**, the right hemisphere surface **(C)**, and an axial view **(D)**. Simple differences from baseline **(E)** to post-treatment in network-wise functional revealed general patterns of increase in the placebo group (**F**, top) and decreases in the DHA + EPA group (**F**, bottom). Baseline-adjusted network models revealed greater connectivity between the FrontoPartietal Control network and the Default Mode Network in the DHA + EPA group compared to the placebo group (*g* = 0.96, *p* = 0.024) **(G)**. Additionally, DHA + EPA was associated with greater regional homogeneity (*g* > 1.07) in the left somatomotor cortex as well as an area of the left retrospenium while greater regional homogeneity (*g* > 0.97) in the placebo group was observed in bilateral areas of the dorsal prefrontal cortex and one area of the left inferior parietal lobule (all FDR corrected *p* < 0.05) **(H)**. Specific region names creating the edges in **(A–D)** are reported in Supplementary Table 3. Specific region names for those identified in **(H)** are reported in Supplementary Table 2.

Because there were observed group differences in structural connectivity over the course of the study, then functional connectivity could be an indicator of preserved communication between spatially remote (inter-regional) and within local (intra-regional) regions. After controlling for baseline edgewise functional connectivity, a total of 14 edges (0.015% of the total 95,266 edges examined) exhibited group-wise differences that survived multiple comparisons correction at an FDR-corrected *p* < 0.01 (|*g*| > 1.48; Figures 5A–D; Supplementary Table 3). Stronger connectivity in the DHA+EPA group was observed at end of season in 9 of these edges, including connections linking nodes of the salience/ventral attention network with limbic nodes as well as sub-cortical gray matter (Figures 5A–D).

Mean network-level functional connectivity was also assessed to further explore the effects of DHA + EPA on inter-regional functional connectivity. Across 55 network means (including 10 within- and 45 cross-network pairings), weighted mean connectivity at baseline ranged from −0.147 (Salience/VentralAttention – DMN cross-network connectivity) to 0.238 (TemporoParietal network) in the placebo group (Figure 5E, upper panel) and −0.165 (Salience/VentralAttention – DMN cross-network connectivity) to 0.182 (TemporoParietal network) in the DHA + EPA group (Figure 5E, lower panel). Simple changes over the season suggested increased connectivity in several network pairs in the placebo group (Figure 5F, upper panel) while decreased or unchanged connectivity in the majority of the network pairs in the DHA + EPA group (Figure 5F, lower panel). After controlling for baseline connectivity, the DHA+EPA group exhibited greater connectivity between the FrontoParietal Control network and the Default Mode Network (*g* = 0.96, 95% CI: 0.12, 1.67, *p* = 0.024; Figure 5G).

After adjusting for baseline regional homogeneity, a total of 6 ROIs exhibited group-wise differences at end of season (|*g*| > 0.97, FDR corrected *p* < 0.05; Figure 5H; Supplementary Table 2). Greater ReHo in the placebo group was observed in bilateral areas of the dorsal prefrontal cortex and one area of the left inferior parietal lobule. In contrast, greater ReHo in the DHA+EPA group was observed in regions of the left hemisphere's somatomotor cortex as well as an area of the left retrospenium.

### Serum NfL is associated with greater mean, axial, and radial diffusivity throughout the brain

**Figure 6:**
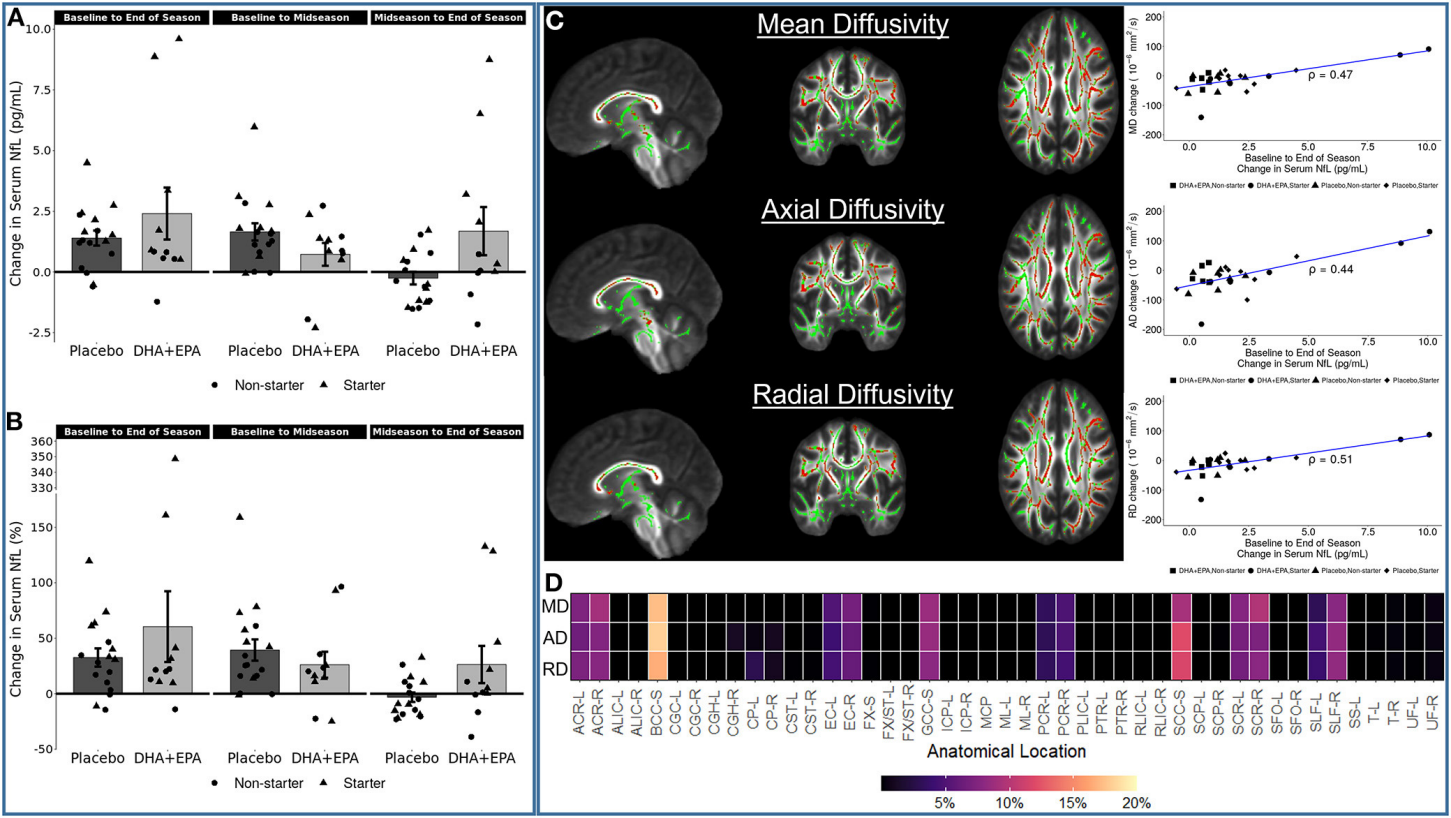
Associations between serum neurofilament light (NfL) change and diffusion tensor metric changes. Serum NfL increased in the majority of participants from baseline to end of season, irrespective of group **(A,B)**. Increasing NfL was positive correlated with mean diffusivity (MD), axial diffusivity (AD), and radial diffusivity (RD) [**(C)**; all family-wise error corrected *p* < 0.05]. Statistically significant voxels were primarily located in the corpus callosum, bilateral corona radiata, and bilateral external capsule **(D)**. The heatmap in **(D)** shows the proportion of statistically significant voxels localized to a given region of interest in the JHU ICBM-DTI-81 atlas. Not all voxels could be assigned to a region based on the spatial extent of the atlas not covering all of the white matter. Region names associated with the abbreviations on the *x*-axis are reported in Supplementary Table 4.

Decline in white matter structural integrity is indicative of axonal damage. To address this possibility, NfL, a blood-based biomarker of axonal damage, was assessed in plasma and subsequently correlated with changes in white matter integrity. NfL concentrations increased from baseline to end of season (Figures 6A,B), with individuals receiving DHA + EPA exhibiting overall greater increases in NfL. Because there were no differences between groups in any of the voxel-wise analyses, correlations between change in NfL levels and both diffusion tensor (FA, MD, AD, RD) and kurtosis (KFA, MKT, AK, RK) metrics were fit for all participants irrespective of group. These analyses revealed that decreased white matter microstructural integrity was associated with increased NfL. Specifically, voxel-wise analyses revealed statistically significant, positive associations between increased NfL and increased mean, axial, and radial diffusivity (Figure 6C). These associations were observed primarily in the corpus callosum as well as bilaterally in the anterior, superior, and posterior corona radiata, external capsule, and the right superior longitudinal fasciculus (Figure 6D).

**Figure 7:**
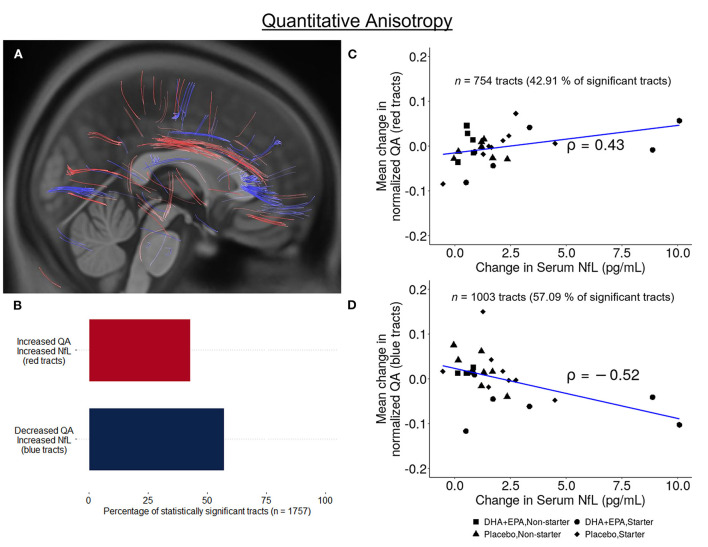
Association between change in serum NfL and white matter structural connectivity. A total of 1,757 tracts exhibited a statistically significant correlation between change in quantitative anisotropy and change in serum neurofilament light (NfL) from baseline to end of season. 42.91% of these tracts exhibited a positive correlation (*n* = 754, FDR corrected *p* < 0.003; **A** red-colored tracts; **(B,C)**] while 57.09% exhibited a negative correlation [*n* = 1,003, FDR corrected *p* < 0.0004; **A** blue-colored tracts; **(B,D)**].

Similarly, increases in NfL over the course of the season were also associated with both increases (*n* = 754 tracts; FDR corrected *p* < 0.002; Figure 7A red tracts, 7B,C) and decreases (*n* = 1,003 tracts; FDR corrected *p* < 0.0004; Figure 7A blue tracts, 7B,D) in QA assessed using deterministic tractography. Positive associations between changes in NfL and QA were observed in the left anterior corticostriatial tract as well as bilateral superior longitudinal fasciculi and thalamic radiations. Increasing NfL and decreasing QA was primarily observed in the corpus callosum and right anterior corticostriatal tract.

## Discussion

In the present study, we present exploratory findings on the effects of DHA + EPA supplementation on neuroimaging biomarkers of repetitive sub-concussive head impacts over the course of a single NCAA football season. Based on prior preclinical and human study reports (29–42), we hypothesized that individuals receiving the specified DHA + EPA treatment regimen, compared to placebo, would exhibit protection against axonal damage resulting from RSHIs measured by indicators of white matter integrity, including fractional anisotropy and mean diffusivity, from baseline to postseason. Based on our results, this hypothesis was not supported. However, secondary indicators of both structural and functional connectivity suggest potentially neuroprotective effects of DHA + EPA supplementation despite evidence of axonal damage.

### NfL is a sensitive measure of white matter damage that may not be ameliorated by omega-3 supplementation

Reduced fractional anisotropy and increased mean diffusivity are commonly reported in studies of RSHIs (20–24). Here we observed no differences between groups for tensor-based metrics, indicating that sustained damage may not have been ameliorated by DHA + EPA supplementation. However, significantly decreased axial diffusivity coupled with non-significantly decreased mean diffusivity, was observed in multiple left hemisphere white matter pathways. Decreased AD is often associated with axonal damage in animal models (91–93) and has been observed in both acute concussion and long term recovery (94, 95).

In contrast, we detected increased diffusivity rates (MD, AD, RD) associated with increased serum NfL (a peripheral biomarker of neuronal damage) from baseline to end of season irrespective of treatment group. The NfL levels observed in the present study are consistent with the range of values observed in previous RSHI studies (<20 pg/ml) (96–98), including the two previous trials examining omega-3 supplementation in this population (38, 39), indicating that even the highest values observed at the end of the season are within an expected range. To date, there have been few studies specifically examining the relationship between NfL and neuroimaging-derived white matter integrity in mTBI or RSHI vulnerable populations (99–102). These studies have identified decreased FA and increased mean diffusivity, particularly in the corpus callosum, to be associated with increased NfL (99, 101, 102). Several of those studies have also identified increased radial diffusivity in similar regions to be associated with increased NfL (99, 100). In line with those findings, we identified increased MD and RD as well as decreased quantitative anisotropy correlated with increased NfL in the corpus callosum as well as more wide-spread positive correlations between NfL and both MD and RD in the anterior, posterior, and superior components of the bilateral corona radiata and bilateral external capsule. Prior work has also identified decreased white matter integrity in the corona radiata following both RSHIs (22, 23) and SRCs (94, 103–108) and the present study reconfirms the susceptibility of these pathways to damage during a single season of football.

We further identified a novel positive correlation between NfL concentration and AD in these same pathways. This relationship has not been identified in prior work examining NfL and diffusion metrics following either RSHIs or SRCs. However, recent studies on mTBIs have shown concomitant increases in mean, axial, and radial diffusivity in patient populations (103, 104, 107–110), and the paired increase in AD and RD associated with serum NfL is consistent with those studies. While the precise nature of the underlying pathology leading to joint increases mean, axial, and radial diffusion metrics is unclear, plausible explanations include axonal swelling (111) or degeneration (92), demyelination (112, 113), and edema (114).

An unanticipated finding of the present study was a cluster of voxels, though not statistically significant, where the mean of the kurtosis tensor increased in the placebo group and decreased in the DHA + EPA treatment group. In animal models of mTBI, reduced increased mean kurtosis was observed throughout the acute and sub-acute recovery phases. The magnitude of increased kurtosis was linked both to proximity to the injury site as well as locally increased reactive astrogliosis (115, 116). Increased mean kurtosis was also observed in a study of RSHIs over a football season (117). Further studies are necessary to fully explain the underlying physiological processes related to increased *in-vivo* mean kurtosis on diffusion weighted imaging in this population.

Broadly, the changes over the course of the season in diffusivity metrics indicate that, in the absence of other explanatory variables (e.g., number of head impacts, prior mTBI history), changes in diffusion likely reflect axonal injury where swelling or edema have resolved. Further, serum NfL may be a sensitive peripheral metric of on-going white matter pathological processes, such as swelling, edema, or degeneration, which have not yet resolved. In this respect, RSHIs may increase the susceptibility of these pathways to further damage and DHA + EPA supplementation, as provided in the present study and to these participants, was insufficient to result in detectably large between-group differences in these changes.

### Omega-3 supplementation preserved local structural connectivity in pathways less susceptible to injury

Further, group-wise differences were evident using quantitative anisotropy (QA) for fiber tracking. Statistically significantly greater increases in QA were observed in the DHA + EPA treatment group compared to the placebo group in ascending fiber groups, while significantly greater increases in QA were observed in the corpus callosum and association corticostriatal fibers in the placebo group compared to the DHA.

QA quantifies restricted diffusion density and is a measure of structural connectivity (67). Greater QA indicates greater connectivity and may reflect white matter compactness and microstructural integrity. The present findings revealed increased connectivity between cortical and subcortical regions in the left hemisphere and decreased connectivity in commissural and primarily right hemispheric longitudinal fiber groups for the DHA+EPA group compared to the placebo group. Prior neuroimaging findings in both mTBI and RSHI identify the corpus callosum and long association pathways to be susceptible to injury (20–24). These findings cumulatively indicate that DHA+EPA supplementation exerted potentially neuroprotective effects in the left hemisphere primarily in ascending and short association corticostriatal fibers, but failed to exert a similar effect in more susceptible commissural and long association fibers.

### Omega-3 supplementation does not protect gray matter during a single season of football

There were no notable effects of DHA+EPA supplementation evident from volumetric analyses. Eight out of 473 ROIs demonstrated between-group differences after adjusting for total brain volume and baseline gray matter, with greater volume observed for the treatment group in only three of these regions (Figure 4B). However, reduced gray matter volume was detected from baseline to end of season across the whole sample in multiple areas, including those identified in the between-group analyses, and localized to the anterior and posterior cingulate, superior and middle frontal gyri, and left superior temporal gyrus, among other regions (Figure 4C). These findings collectively suggest that, while individual parcels may have been more or less influenced by treatment group membership, the overarching effect was decreased volume that was not driven by group membership but rather participation during the season. Prior work from sports related concussions, and mild traumatic brain injuries more broadly, indicate reductions in both cortical thickness and volume (115, 118–121). One prior study reported decreased volume following RSHIs (122) over the course of a single season in football players with high impact exposure compared to those with low impact exposure as measured by helmet accelerometry. The present findings agree with these studies and provide further evidence for the detrimental effect of a single season of football on gray matter.

### Omega-3 supplementation preserves connectivity between the default mode and fronto-parietal control networks

Secondary analyses identified greater local, inter-regional, and cross-network functional connectivity in the DHA+EPA treatment group. These differences were large in magnitude and included both local regions and network-wise connections associated with cognitive control. Studies on mTBI have previously demonstrated reduced connectivity between the default mode network and cognitive control networks that may underpin acute cognitive dysregulation and memory disruption (123–125). Here we observed greater connectivity between these two networks after controlling for baseline connectivity in the DHA + EPA treatment group compared to the placebo group. Prior work demonstrates that fatty acid intake, including DHA, is associated with functional network strength during both development (126) and aging (127, 128). Interestingly, only two of the edges demonstrating a between-groups difference involved a region identified to also be significantly different between groups for the gray matter analyses (RH_SomMotA_8). Further, only two ROIs with statistically significant between-group differences in gray matter volume were associated with the identified networks (LH_ContA_Cinga_1, RH_DefaultC_Rsp_1). However, nearly all of the ROIs in the default mode and control networks exhibited decreased gray matter volume from baseline to end of season. Thus, the present findings support the potential for supplementation to preserve functional connectivity between these networks following RSHIs, thus supporting cognitive function, despite evidence of axonal damage and changes in gray matter volume.

### Limitations

There are several critical limitations to these exploratory analyses. First, head impact exposure was not quantified over the course of the study. Starter status was used as a proxy for exposure, as in the Oliver et al. study (38), and the groups here were initially balanced between starters and non-starters. However, this proxy measure may be insufficient to quantify the density of RSHIs sustained during a football season. Assessment that directly quantifies head impact density and magnitude during the season is necessary to fully understand the potential role of DHA + EPA supplementation as well as to draw direct correlations with serum NfL.

Secondly, this study had a small sample size. This trial was powered on NfL change as the primary outcome (43). For neuroimaging analyses, sample size calculations indicated sufficient power to detect an approximately 10% difference between groups in FA based on 19 per group as initially randomized. However, eight individuals in the treatment group dropped out or were excluded, which substantially decreased our statistical power to detect differences as small as this. Prior trials on omega-3 supplementation in American football have indicated reduced serum NfL levels compared to controls by the end of the season, particularly in starters, which were not observed here and the considerably smaller sample size likely contributed to these differences (38, 39).

To partially address the sample size concerns, all analyses employed permutation-based estimations of empirical *p*-values and bootstrap estimations of effect size confidence intervals for region-based analyses and, particularly for voxelwise analyses, smallest observed p-values are reported. While these methods do not fully overcome the challenges of small samples, they do provide information to guide future trial planning.

Third, recent evidence published after this trial was completed suggests that oleic acid may exert neuroprotective effects in cerebral ischemia models (129), and therefore may have exerted an unplanned positive effect in the placebo group. Fourth, it was not possible to time-lock blood draws and MRI acquisition. All draws were completed in the same day to ensure time-locking of blood-based markers; however imaging occurred over a three-week (baseline) and two-week (end of season) window. Therefore, associations between imaging and NfL reported here may not fully reflect the true relationship between these measures. NfL levels remain elevated up to several days after a concussive event (130), while the emphasis here is on repetitive, subconcussive injuries that occur on a daily basis due to practice and game participation. As such, the NfL levels and relationships reported here may not sufficiently capture a “steady state” level of NfL associated with these micro-traumatic events throughout the season. Finally, no injury history data were available, and past history of SRCs may have contributed to the present findings.

## Future directions

The outcomes from the present study along with the identified limitations suggest the opportunity for further exploration. Future studies should include measures of head impact density and magnitude during the season along with injury history in order to better quantify head impacts above and beyond starter status and field position. Additionally, there is the need for work comparing omega-3 supplementation and placebo in individuals sustaining RSHIs with non-contact controls in order to better characterize changes in neuroimaging metrics resulting from football participation and affected by supplementation. Finally, there is the need to connect neuroimaging and serum outcomes with cognitive and behavioral performance to provide evidence of improved or maintained function with omega-3 supplementation.

## Conclusions

The present study is the first to examine the association between DHA + EPA supplementation, neuroimaging biomarkers and serum NfL in American football players. A single season of collegiate football was associated with multiple indicators of white matter microstructural damage which were correlated with elevated NfL blood levels. Findings of locally preserved quantitative anisotropy (structural connectivity) and cross-network functional connectivity suggests that DHA + EPA supplementation may exert neuroprotective effects despite evidence of white matter damage in other regions. Further large-scale studies that include neuroimaging and direct quantification of head impact density could inform the potential of DHA + EPA as a preventive approach, and should include testing alternative formulations, doses, and regimens. The question of whether DHA+EPA could promote repair of brain damage in this population remains to be established.

## Data availability statement

The raw data supporting the conclusions of this article will be made available by the authors, without undue reservation.

## Ethics statement

The studies involving human participants were reviewed and approved by Institutional Review Board of the University of Arizona. The patients/participants provided their written informed consent to participate in this study.

## Author contributions

VM and FC concept, execution, analysis of blood-based biomarkers, and manuscript revision. GH, YW, and CL trial oversight, interpretation, drafting, revision. AR and WK neuroimaging analyses, data harmonization, interpretation, drafting, and revision. All authors have contributed substantially to the manuscript and read and agreed to the published version of the manuscript.

## Funding

Conduct of the study was supported by the Center for Innovation in Brain Science, University of Arizona, Tucson AZ (https://cibs.uahs.arizona.edu/).

## Conflict of interest

Author FC is a founder of Tyrion Omega LLC. Authors GH, CL, and RB hold leadership positions in NEUTherapeutics. The remaining authors declare that the research was conducted in the absence of any commercial or financial relationships that could be construed as a potential conflict of interest.

## Publisher's note

All claims expressed in this article are solely those of the authors and do not necessarily represent those of their affiliated organizations, or those of the publisher, the editors and the reviewers. Any product that may be evaluated in this article, or claim that may be made by its manufacturer, is not guaranteed or endorsed by the publisher.
