# Supplementary material for: Effects of docosahexaenoic acid and eicosapentaoic acid supplementation on white matter integrity after repetitive sub-concussive head impacts during American football: Exploratory neuroimaging findings from a pilot RCT
Source: Front Neurol. 2022 Sep 15;13:891531. doi: 10.3389/fneur.2022.891531 (PMC9521411; doi:10.3389/fneur.2022.891531)
Supplement: Supplementary file 1 [file Data_Sheet_1.docx]

Supplemental Table 1. CONSORT Checklist

| Section/Topic | Item No | Checklist item | Reported on page No |
| --- | --- | --- | --- |
| Title and abstract | | | |
|  | 1a | Identification as a randomised trial in the title | 1 |
|  | 1b | Structured summary of trial design, methods, results, and conclusions (for specific guidance see CONSORT for abstracts) | 2 |
| Introduction | | | |
| Background and objectives | 2a | Scientific background and explanation of rationale | 3 |
|  | 2b | Specific objectives or hypotheses | 4 |
| Methods | | | |
| Trial design | 3a | Description of trial design (such as parallel, factorial) including allocation ratio | 5 |
|  | 3b | Important changes to methods after trial commencement (such as eligibility criteria), with reasons |  |
| Participants | 4a | Eligibility criteria for participants | 5 |
|  | 4b | Settings and locations where the data were collected | 5 |
| Interventions | 5 | The interventions for each group with sufficient details to allow replication, including how and when they were actually administered | 6 |
| Outcomes | 6a | Completely defined pre-specified primary and secondary outcome measures, including how and when they were assessed | Prior publication, pg 5 |
|  | 6b | Any changes to trial outcomes after the trial commenced, with reasons | Prior publication |
| Sample size | 7a | How sample size was determined | Prior publication |
|  | 7b | When applicable, explanation of any interim analyses and stopping guidelines | NA |
| Randomisation: |  |  |  |
| Sequence generation | 8a | Method used to generate the random allocation sequence | 5 |
|  | 8b | Type of randomisation; details of any restriction (such as blocking and block size) | 5 |
| Allocation concealment mechanism | 9 | Mechanism used to implement the random allocation sequence (such as sequentially numbered containers), describing any steps taken to conceal the sequence until interventions were assigned | 5 |
| Implementation | 10 | Who generated the random allocation sequence, who enrolled participants, and who assigned participants to interventions | Prior publication |
| Blinding | 11a | If done, who was blinded after assignment to interventions (for example, participants, care providers, those assessing outcomes) and how | 5 |
|  | 11b | If relevant, description of the similarity of interventions | 5 |
| Statistical methods | 12a | Statistical methods used to compare groups for primary and secondary outcomes |  |
|  | 12b | Methods for additional analyses, such as subgroup analyses and adjusted analyses | 11-13 |
| Results | | | |
| Participant flow (a diagram is strongly recommended) | 13a | For each group, the numbers of participants who were randomly assigned, received intended treatment, and were analysed for the primary outcome | 14 |
|  | 13b | For each group, losses and exclusions after randomisation, together with reasons | Figure 1 |
| Recruitment | 14a | Dates defining the periods of recruitment and follow-up | 5 |
|  | 14b | Why the trial ended or was stopped | 5 |
| Baseline data | 15 | A table showing baseline demographic and clinical characteristics for each group | Table 1-2 |
| Numbers analysed | 16 | For each group, number of participants (denominator) included in each analysis and whether the analysis was by original assigned groups | 14 |
| Outcomes and estimation | 17a | For each primary and secondary outcome, results for each group, and the estimated effect size and its precision (such as 95% confidence interval) | Prior publication, for exploratory analyses: 14-16 |
|  | 17b | For binary outcomes, presentation of both absolute and relative effect sizes is recommended | NA |
| Ancillary analyses | 18 | Results of any other analyses performed, including subgroup analyses and adjusted analyses, distinguishing pre-specified from exploratory | 14-16 |
| Harms | 19 | All important harms or unintended effects in each group (for specific guidance see CONSORT for harms) | Prior publication |
| Discussion | | | |
| Limitations | 20 | Trial limitations, addressing sources of potential bias, imprecision, and, if relevant, multiplicity of analyses | 19-20 |
| Generalisability | 21 | Generalisability (external validity, applicability) of the trial findings | Prior publication |
| Interpretation | 22 | Interpretation consistent with results, balancing benefits and harms, and considering other relevant evidence | 17-19 |
| Other information | | |  |
| Registration | 23 | Registration number and name of trial registry | 2 |
| Protocol | 24 | Where the full trial protocol can be accessed, if available | NA |
| Funding | 25 | Sources of funding and other support (such as supply of drugs), role of funders | 20-21 |

| Supplemental Table 2. Region names, effect sizes, and interpretations for treatment-related between group differences in gray matter volume (Figure 2A-B) and regional homogeneity (Figure 5H) | | | |
| --- | --- | --- | --- |
| Region name | Atlas | Hedges *g* | Interpretation |
| ***Baseline differences in gray matter volume (Figure 2A)*** | | | |
| RH_SomMotA_14 | Schaefer 400x17 | -1.099 | Placebo > DHA+EPA |
| RH_ContA_PFCl_2 | Schaefer 400x17 | -1.063 | Placebo > DHA+EPA |
| RH_DorsAttnA_SPL_1 | Schaefer 400x17 | -1.041 | Placebo > DHA+EPA |
| RH_ContA_PFCl_5 | Schaefer 400x17 | -1.000 | Placebo > DHA+EPA |
| RH_SomMotA_16 | Schaefer 400x17 | 0.780 | DHA+EPA > Placebo |
| LH_SomMotB_Aud_9 | Schaefer 400x17 | 0.865 | DHA+EPA > Placebo |
| RH_Limbic_OFC_5 | Schaefer 400x17 | 0.896 | DHA+EPA > Placebo |
| LH_SomMotA_2 | Schaefer 400x17 | 0.917 | DHA+EPA > Placebo |
| LH_DefaultA_PCC_5 | Schaefer 400x17 | 1.082 | DHA+EPA > Placebo |
| LH_DefaultB_PFCd_2 | Schaefer 400x17 | 1.197 | DHA+EPA > Placebo |
| ***Baseline-adjusted end of season differences in gray matter volume (Figure 2B)*** | | | |
| LH_ContA_Cinga_1 | Schaefer 400x17 | -1.521 | Placebo > DHA+EPA |
| LH_SomMotB_Aud_2 | Schaefer 400x17 | -1.156 | Placebo > DHA+EPA |
| RH_SomMotA_8 | Schaefer 400x17 | -1.095 | Placebo > DHA+EPA |
| LH_SomMotB_Aud_8 | Schaefer 400x17 | -0.981 | Placebo > DHA+EPA |
| RH_SalVentAttnA_ParMed_6 | Schaefer 400x17 | -0.920 | Placebo > DHA+EPA |
| LH_VisPeri_ExStrSup_2 | Schaefer 400x17 | 1.472 | DHA+EPA > Placebo |
| RH_DefaultC_Rsp_1 | Schaefer 400x17 | 1.078 | DHA+EPA > Placebo |
| RH_SomMotB_S2_8 | Schaefer 400x17 | 1.000 | DHA+EPA > Placebo |
| ***Baseline difference in regional homogeneity (not pictured)*** | | | |
| LH_SalVentAttnA_FrMed_1 | Schaefer 400x17 | -1.249 | Placebo > DHA+EPA |
| RH_SomMotB_S2_11 | Schaefer 400x17 | -1.170 | Placebo > DHA+EPA |
| RH_SomMotA_8 | Schaefer 400x17 | 0.973 | DHA+EPA > Placebo |
| LH_ContB_IPL_1 | Schaefer 400x17 | 1.028 | DHA+EPA > Placebo |
| ***Baseline-adjusted end of season differences in regional homogeneity (Figure 5H)*** | | | |
| LH_ContB_IPL_2 | Schaefer 400x17 | -1.487 | Placebo > DHA+EPA |
| LH_DefaultB_PFCd_2 | Schaefer 400x17 | -1.023 | Placebo > DHA+EPA |
| RH_DefaultB_PFCd_4 | Schaefer 400x17 | -0.970 | Placebo > DHA+EPA |
| LH_SomMotB_Aud_6 | Schaefer 400x17 | 0.945 | DHA+EPA > Placebo |
| Cerebellar #5 | Buckner | 1.066 | DHA+EPA > Placebo |
| LH_SomMotA_6 | Schaefer 400x17 | 1.072 | DHA+EPA > Placebo |
| LH_DefaultC_Rsp_3 | Schaefer 400x17 | 1.107 | DHA+EPA > Placebo |
| Notes: Region names are presented as defined in the respective atlases. The Schaefer 400x17 atlas (Schaefer et al., 2018; <https://dx.doi.org/10.1093/cercor/bhx179>) embeds is a 400 cortical region atlas with integration of Yeo 17-network atlas (Yeo et al., 2011; <https://doi.org/10.1152%2Fjn.00338.2011>) to assign regions to networks. The Buckner cerebellar atlas (Buckner et al., 2011; <https://doi.org/10.1152%2Fjn.00339.2011>) includes a total of 37 regions. | | | |

| Supplemental Table 3. Edge node names, effect sizes, and interpretations for treatment-related between group differences in edgewise-functional connectivity (Figure 5A-D) | | | |
| --- | --- | --- | --- |
| Node #1 Name | Node #2 Name | Hedges *g* | Interpretation |
| LH_ContC_pCun_2 | RH_SalVentAttnA_Ins_2 | -1.952 | Placebo > DHA+EPA |
| RH_SomMotA_8 | R. Ventromedial Putamen* | -1.808 | Placebo > DHA+EPA |
| RH_SomMotA_8 | R. Dorsolateral Putamen* | -1.800 | Placebo > DHA+EPA |
| LH_DorsAttnB_PostC_6 | RH_ContC_pCun_2 | -1.775 | Placebo > DHA+EPA |
| LH_ContC_Cingp_2 | RH_DefaultA_PFCd_1 | -1.687 | Placebo > DHA+EPA |
| LH_SomMotB_Aud_1 | RH_ContB_PFCld_3 | -1.503 | Placebo > DHA+EPA |
| RH_SomMotB_S2_5 | RH_SalVentAttnB_PFCl_2 | -1.481 | Placebo > DHA+EPA |
| RH_ContB_IPL_2 | RH_DefaultC_Rsp_2 | 1.583 | DHA+EPA > Placebo |
| LH_SalVentAttnB_PFCmp_1 | RH_Limbic_TempPole_1 | 1.625 | DHA+EPA > Placebo |
| LH_SalVentAttnA_FrMed_2 | LH_Limbic_TempPole_1 | 1.642 | DHA+EPA > Placebo |
| LH_SalVentAttnB_PFCl_3 | LH_DefaultB_Temp_4 | 1.657 | DHA+EPA > Placebo |
| LH_Limbic_TempPole_7 | RH_Limbic_OFC_6 | 1.681 | DHA+EPA > Placebo |
| RH_SalVentAttnA_ParMed_4 | R. Rostral Hippocampus* | 1.712 | DHA+EPA > Placebo |
| LH_DorsAttnB_FEF_2 | LH_DefaultB_PFCd_3 | 1.724 | DHA+EPA > Placebo |
| RH_SalVentAttnA_ParMed_4 | L. Lateral Amygdala* | 1.872 | DHA+EPA > Placebo |
| Notes: Regions denoted by a “*” come from the Brainnetome subcortical atlas (Fan et al., 2016; <https://doi.org/10.1093/cercor/bhw157>). All other regions come from the Schaefer 400x17 atlas (Schaefer et al., 2018; <https://dx.doi.org/10.1093/cercor/bhx179>). Differences here represent the difference between groups in baseline-adjusted functional connectivity in the edge defined by the two nodes. | | | |

| Supplementary Table 4. JHU ICBM-DTI-81 White-Matter Labels atlas abbreviations depicted in Figure 6D | | | | | |
| --- | --- | --- | --- | --- | --- |
| MCP | Middle cerebellar peduncle (Bilateral) | ALIC-L | Anterior limb of the internal capsule (Left) | EC-L | External capsule (Left) |
| GCC-S | Genu of corpus callosum (Bilateral) | PLIC-L | Posterior limb of the internal capsule (Right) | CGC-R | Cingulate gyrus (Right) |
| BCC-S | Body of corpus callosum (Bilateral) | PLIC-L | Posterior limb of the internal capsule (Left) | CGC-L | Cingulate gyrus (Left) |
| SCC-S | Splenium of corpus callosum (Bilateral) | RLIC-R | Retrolenticular part of the internal capsule (Right) | CGH-R | Cingulum (hippocampus) (Right) |
| FX-S | Fornix (column and body of fornix) (Bilateral) | RLIC-L | Retrolenticular part of the internal capsule (Left) | CGH-L | Cingulum (hippocampus) (Left) |
| CST-R | Corticospinal tract (Right) | ACR-R | Anterior corona radiata (Right) | FX/ST-R | Fornix (cres) / Stria terminalis (Right) |
| CST-L | Corticospinal tract (Left) | ACR-L | Anterior corona radiata (Left) | FX/ST-L | Fornix (cres) / Stria terminalis (Left) |
| ML-R | Medial lemniscus (Right) | SCR-R | Superior corona radiata (Right) | SLF-R | Superior longitudinal fasciculus (Right) |
| ML-L | Medial lemniscus (Left) | SCR-L | Superior corona radiata (Left) | SLF-L | Superior longitudinal fasciculus (Left) |
| ICP-R | Inferior cerebellar peduncle (Right) | PCR-R | Posterior corona radiata (Right) | SFO-R | Superior fronto-occipital fasciculus (Right) |
| ICP-L | Inferior cerebellar peduncle (Left) | PCR-L | Posterior corona radiata (Left) | SFO-L | Superior fronto-occipital fasciculus (Left) |
| SCP-R | Superior cerebellar peduncle (Right) | PTR-R | Posterior thalamic radiation (Right) | UF-R | Uncinate fasciculus (Right) |
| SCP-L | Superior cerebellar peduncle (Left) | PTR-L | Posterior thalamic radiation (Left) | UF-L | Uncinate fasciculus (Left) |
| CP-R | Cerebral peduncle (Right) | SS-L | Sagittal stratum (Right) | T-R | Tapetum (Right) |
| CP-L | Cerebral peduncle (Left) | SS-L | Sagittal stratum (Left) | T-L | Tapetum (Left) |
| ALIC-R | Anterior limb of the internal capsule (Right) | EC-R | External capsule (Right) |  |  |
| Note: Anatomical label abbreviations for the JHU-ICBM-DTI-81 atlas (Oishi et al, 2008; <https://doi.org/10.1016/j.neuroimage.2008.07.009>) | | | | | |
